# Metronidazolium perchlorate

**DOI:** 10.1107/S1600536810038055

**Published:** 2010-09-30

**Authors:** Yong-Tao Wang, Xiao-Lei Chu, Shi-Chen Yan, Gui-Mei Tang

**Affiliations:** a Shandong Provincial Key Laboratory of Fine Chemicals, Department of Chemical Engineering, Shandong Institute of Light Industry, Jinan, Shandong 250353, People’s Republic of China

## Abstract

In the crystal structure of the title compound [systematic name: 1-(2-hy­droxy­eth­yl)-2-methyl-5-nitro-1*H*-imidazol-3-ium perchlorate], C_6_H_10_N_3_O_3_^+^·ClO_4_^−^, the cations are linked by inter­molecular N—H⋯O hydrogen bonds into zigzag chains along the *c* axis. The cations and anions are connected by O—H⋯O and C—H⋯O hydrogen bonds. A weak intra­molecular C—H⋯O hydrogen bond is also observed.

## Related literature

For metronidazole, see: Castelli *et al.* (2000 ▶); Contrerasa *et al.* (2009 ▶). For a related structure, see: Wang *et al.* (2006 ▶).
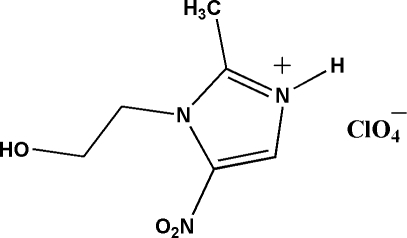


## Experimental

### 

#### Crystal data

C_6_H_10_N_3_O_3_^+^·ClO_4_^−^
*M* *_r_* = 271.62
Monoclinic, 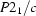

*a* = 7.8541 (13) Å
*b* = 10.6791 (17) Å
*c* = 13.032 (2) Å
β = 93.904 (2)°
*V* = 1090.5 (3) Å^3^
*Z* = 4
Mo *K*α radiation
μ = 0.38 mm^−1^
*T* = 296 K
0.40 × 0.20 × 0.20 mm

#### Data collection

Bruker SMART CCD area-detector diffractometer
Absorption correction: multi-scan (*SADABS*; Sheldrick, 1996 ▶) *T*_min_ = 0.862, *T*_max_ = 0.928
9191 measured reflections
2509 independent reflections
2219 reflections with *I* > 2σ(*I*)
*R*_int_ = 0.030

#### Refinement

*R*[*F* ^2^ > 2σ(*F* ^2^)] = 0.053
*wR*(*F* ^2^) = 0.159
*S* = 1.04
2509 reflections
155 parameters
H-atom parameters constrained
Δρ_max_ = 0.60 e Å^−3^
Δρ_min_ = −0.43 e Å^−3^

### 

Data collection: *SMART* (Bruker, 2001 ▶); cell refinement: *SAINT* (Bruker, 2001 ▶); data reduction: *SAINT*; program(s) used to solve structure: *SHELXS97* (Sheldrick, 2008 ▶); program(s) used to refine structure: *SHELXL97* (Sheldrick, 2008 ▶); molecular graphics: *SHELXTL* (Sheldrick, 2008 ▶); software used to prepare material for publication: *SHELXTL*.

## Supplementary Material

Crystal structure: contains datablocks global, I. DOI: 10.1107/S1600536810038055/is2601sup1.cif

Structure factors: contains datablocks I. DOI: 10.1107/S1600536810038055/is2601Isup2.hkl

Additional supplementary materials:  crystallographic information; 3D view; checkCIF report

## Figures and Tables

**Table 1:** Hydrogen-bond geometry (Å, °)

*D*—H⋯*A*	*D*—H	H⋯*A*	*D*⋯*A*	*D*—H⋯*A*
O1—H1⋯O5	0.89	2.02	2.860 (4)	157
N2—H2⋯O1^i^	0.83	1.98	2.803 (3)	169
C1—H1*B*⋯O2	0.97	2.52	3.126 (3)	121
C6—H6*B*⋯O7^i^	0.96	2.52	3.441 (4)	161

Symmetry code: (i) 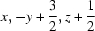.

**Fig. 1.**
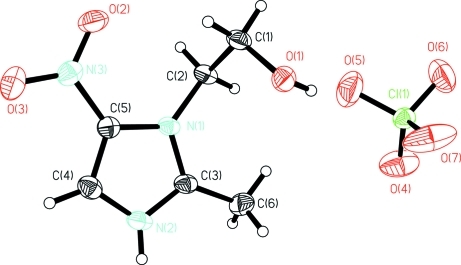
The molecular structure of (I), with the atom numbering scheme. Displacement ellipsoids are drawn at the 30% probability level.

**Fig. 2.**
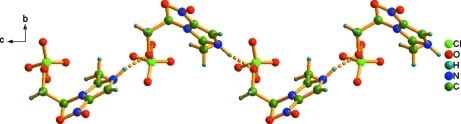
One-dimensional chain running along the c axis.

## supplementary crystallographic information

### Comment

Metronidazole is usually applied in the area of anaerobic protozoan and bacterial infections (Castelli *et al.*, 2000). Its solubility is low in water, so that its absorption is not easy in human body. To solve this problem and to increase its solubility in water, a kind of new strategy of protonated metronidazole has been studied though other methods have been developed in the area of medicine, for example, metal complexes (Contrerasa *et al.*, 2009) and pharmaceutical co-crystals. However, co-crystals containing metronidazole has rarely been investigated. In this paper, we report the 1:1 salt formed by metronidazole and perchloric acid, (I).

A view of the title structure is shown in Fig. 1. The H atom is transferred from the perchloric acid group to the imidazole N atom forming an 1:1 organic salt, which is similar to other organic salt published previously (Wang *et al.*, 2006). In the crystal structure, one-dimensional chains are formed *via* intermolecular O—H···O and N—H···O hydrogen bonds (Table 1 and Fig. 2).

### Experimental

Metronidazole (1.71 g, 10 mmol) and 75% aqueous HClO_4_ (2 ml) were mixed and dissolved in 10 ml water. The reaction mixture was stirred slowly to room temperature. The bar colourless crystals suitable for X-ray diffraction were obtained after two weeks. Analysis found: C 26.17, H 3.69, N 15.41%; calcd. : C 26.53, H 3.71, N 15.47%. IR (KBr, cm^-1^): 3394, 3078, 1610, 1546, 1527, 1502, 1411, 1373, 1319, 1251, 1193, 1143, 1111, 1085, 1080, 1062, 037, 867, 831, 736, 671, 630, 559, 516.

### Refinement

All H atoms were located in a difference Fourier map. Oxygen- and nitrogen-bound H atoms were then refined as riding, with *U*_iso_(H) = 1.5*U*_eq_(O, N). Carbon-bound H atoms were positioned geometrically (C—H = 0.96 or 0.97 Å), and were included in the refinement in the riding-model approximation, with *U*_iso_(H) = 1.2*U*_eq_(C).

### Crystal data

**Table:**

C_6_H_10_N_3_O_3_^+^·ClO_4_^−^	*F*(000) = 560
*M**_r_* = 271.62	*D*_x_ = 1.654 Mg m^−^^3^
Monoclinic, *P*2_1_/*c*	Mo *K*α radiation, λ = 0.71073 Å
Hall symbol: -P 2ybc	Cell parameters from 5428 reflections
*a* = 7.8541 (13) Å	θ = 2.5–27.5°
*b* = 10.6791 (17) Å	µ = 0.38 mm^−^^1^
*c* = 13.032 (2) Å	*T* = 296 K
β = 93.904 (2)°	Prism, colourless
*V* = 1090.5 (3) Å^3^	0.40 × 0.20 × 0.20 mm
*Z* = 4	

### Data collection

**Table:**

Bruker SMART CCD area-detector diffractometer	2509 independent reflections
Radiation source: fine-focus sealed tube	2219 reflections with *I* > 2σ(*I*)
graphite	*R*_int_ = 0.030
φ and ω scans	θ_max_ = 27.5°, θ_min_ = 2.5°
Absorption correction: multi-scan (*SADABS*; Sheldrick, 1996)	*h* = −10→10
*T*_min_ = 0.862, *T*_max_ = 0.928	*k* = −13→13
9191 measured reflections	*l* = −16→16

### Refinement

**Table:**

Refinement on *F*^2^	Secondary atom site location: difference Fourier map
Least-squares matrix: full	Hydrogen site location: inferred from neighbouring sites
*R*[*F*^2^ > 2σ(*F*^2^)] = 0.053	H-atom parameters constrained
*wR*(*F*^2^) = 0.159	*w* = 1/[σ^2^(*F*_o_^2^) + (0.085*P*)^2^ + 0.8145*P*] where *P* = (*F*_o_^2^ + 2*F*_c_^2^)/3
*S* = 1.04	(Δ/σ)_max_ = 0.001
2509 reflections	Δρ_max_ = 0.60 e Å^−^^3^
155 parameters	Δρ_min_ = −0.43 e Å^−^^3^
0 restraints	Extinction correction: *SHELXL97* (Sheldrick, 2008), Fc^*^=kFc[1+0.001xFc^2^λ^3^/sin(2θ)]^-1/4^
Primary atom site location: structure-invariant direct methods	Extinction coefficient: 0.190 (12)

### Special details

**Table:**

Geometry. All e.s.d.'s (except the e.s.d. in the dihedral angle between two l.s. planes) are estimated using the full covariance matrix. The cell e.s.d.'s are taken into account individually in the estimation of e.s.d.'s in distances, angles and torsion angles; correlations between e.s.d.'s in cell parameters are only used when they are defined by crystal symmetry. An approximate (isotropic) treatment of cell e.s.d.'s is used for estimating e.s.d.'s involving l.s. planes.
Refinement. Refinement of *F*^2^ against ALL reflections. The weighted *R*-factor *wR* and goodness of fit *S* are based on *F*^2^, conventional *R*-factors *R* are based on *F*, with *F* set to zero for negative *F*^2^. The threshold expression of *F*^2^ > σ(*F*^2^) is used only for calculating *R*-factors(gt) *etc*. and is not relevant to the choice of reflections for refinement. *R*-factors based on *F*^2^ are statistically about twice as large as those based on *F*, and *R*- factors based on ALL data will be even larger.

### Fractional atomic coordinates and isotropic or equivalent isotropic displacement parameters (Å^2^)

**Table:**

	*x*	*y*	*z*	*U*_iso_*/*U*_eq_	
O1	0.7469 (2)	0.70762 (17)	0.58597 (12)	0.0486 (4)	
H1	0.8599	0.7118	0.5945	0.073*	
O2	0.4144 (3)	0.4116 (2)	0.66132 (15)	0.0639 (6)	
O3	0.2449 (3)	0.4469 (2)	0.78337 (19)	0.0727 (6)	
N1	0.6726 (2)	0.55501 (15)	0.76908 (12)	0.0340 (4)	
N2	0.6393 (3)	0.64689 (18)	0.91461 (14)	0.0437 (5)	
H2	0.6580	0.6952	0.9643	0.066*	
N3	0.3813 (3)	0.45742 (19)	0.74353 (16)	0.0487 (5)	
C1	0.7048 (3)	0.5773 (2)	0.58035 (16)	0.0451 (5)	
H1A	0.7611	0.5397	0.5240	0.054*	
H1B	0.5827	0.5686	0.5656	0.054*	
C2	0.7567 (3)	0.5070 (2)	0.67880 (16)	0.0404 (5)	
H2A	0.7283	0.4191	0.6694	0.048*	
H2B	0.8794	0.5131	0.6922	0.048*	
C3	0.7495 (3)	0.6272 (2)	0.84260 (15)	0.0384 (5)	
C4	0.4896 (3)	0.5882 (2)	0.88970 (17)	0.0441 (5)	
H4A	0.3928	0.5879	0.9270	0.053*	
C5	0.5098 (3)	0.53022 (19)	0.79949 (16)	0.0379 (5)	
C6	0.9254 (3)	0.6762 (3)	0.8466 (2)	0.0550 (6)	
H6A	0.9791	0.6507	0.7860	0.083*	
H6B	0.9887	0.6439	0.9065	0.083*	
H6C	0.9228	0.7660	0.8500	0.083*	
Cl1	1.22616 (7)	0.74741 (5)	0.59693 (4)	0.0442 (3)	
O4	1.3343 (4)	0.7477 (2)	0.6879 (2)	0.0994 (10)	
O5	1.1033 (4)	0.6500 (3)	0.5984 (3)	0.1022 (10)	
O6	1.3237 (5)	0.7180 (3)	0.5116 (2)	0.1074 (11)	
O7	1.1515 (5)	0.8663 (3)	0.5795 (2)	0.1214 (14)	

### Atomic displacement parameters (Å^2^)

**Table:**

	*U*^11^	*U*^22^	*U*^33^	*U*^12^	*U*^13^	*U*^23^
O1	0.0558 (10)	0.0482 (10)	0.0408 (8)	−0.0064 (7)	−0.0038 (7)	0.0107 (7)
O2	0.0773 (14)	0.0622 (12)	0.0503 (11)	−0.0159 (10)	−0.0096 (9)	−0.0110 (9)
O3	0.0564 (12)	0.0751 (14)	0.0870 (16)	−0.0189 (10)	0.0074 (11)	0.0038 (12)
N1	0.0444 (9)	0.0313 (8)	0.0258 (8)	0.0031 (7)	−0.0008 (6)	0.0019 (6)
N2	0.0642 (12)	0.0368 (9)	0.0302 (9)	0.0026 (8)	0.0033 (8)	−0.0042 (7)
N3	0.0558 (12)	0.0405 (10)	0.0487 (11)	−0.0059 (9)	−0.0055 (9)	0.0065 (8)
C1	0.0627 (14)	0.0457 (12)	0.0266 (9)	−0.0026 (10)	0.0012 (9)	−0.0006 (8)
C2	0.0534 (12)	0.0381 (11)	0.0297 (9)	0.0067 (9)	0.0044 (8)	−0.0019 (8)
C3	0.0510 (12)	0.0356 (10)	0.0279 (9)	0.0016 (8)	−0.0038 (8)	0.0012 (7)
C4	0.0549 (13)	0.0390 (11)	0.0391 (11)	0.0050 (9)	0.0086 (9)	0.0025 (9)
C5	0.0452 (11)	0.0335 (10)	0.0347 (10)	0.0013 (8)	−0.0006 (8)	0.0037 (8)
C6	0.0545 (14)	0.0615 (16)	0.0476 (13)	−0.0111 (12)	−0.0074 (10)	−0.0048 (11)
Cl1	0.0468 (4)	0.0427 (4)	0.0425 (4)	−0.0003 (2)	−0.0022 (2)	−0.0002 (2)
O4	0.126 (2)	0.0840 (18)	0.0799 (17)	0.0238 (15)	−0.0504 (17)	−0.0069 (13)
O5	0.0761 (16)	0.097 (2)	0.137 (3)	−0.0317 (15)	0.0297 (16)	−0.0074 (18)
O6	0.137 (3)	0.106 (2)	0.0864 (19)	−0.013 (2)	0.0566 (19)	0.0002 (16)
O7	0.157 (3)	0.0654 (16)	0.129 (2)	0.0509 (17)	−0.084 (2)	−0.0367 (15)

### Geometric parameters (Å, °)

**Table:**

O1—C1	1.431 (3)	C1—H1B	0.9700
O1—H1	0.8881	C2—H2A	0.9700
O2—N3	1.222 (3)	C2—H2B	0.9700
O3—N3	1.227 (3)	C3—C6	1.475 (3)
N1—C3	1.341 (3)	C4—C5	1.348 (3)
N1—C5	1.390 (3)	C4—H4A	0.9300
N1—C2	1.479 (3)	C6—H6A	0.9600
N2—C3	1.336 (3)	C6—H6B	0.9600
N2—C4	1.353 (3)	C6—H6C	0.9600
N2—H2	0.8328	Cl1—O4	1.410 (3)
N3—C5	1.434 (3)	Cl1—O7	1.411 (2)
C1—C2	1.518 (3)	Cl1—O5	1.420 (3)
C1—H1A	0.9700	Cl1—O6	1.427 (3)
			
C1—O1—H1	106.3	N2—C3—N1	108.12 (19)
C3—N1—C5	106.50 (17)	N2—C3—C6	124.7 (2)
C3—N1—C2	124.35 (18)	N1—C3—C6	127.2 (2)
C5—N1—C2	129.02 (18)	C5—C4—N2	105.7 (2)
C3—N2—C4	110.65 (18)	C5—C4—H4A	127.2
C3—N2—H2	123.8	N2—C4—H4A	127.2
C4—N2—H2	125.3	C4—C5—N1	109.1 (2)
O2—N3—O3	125.3 (2)	C4—C5—N3	124.9 (2)
O2—N3—C5	118.6 (2)	N1—C5—N3	126.03 (19)
O3—N3—C5	116.1 (2)	C3—C6—H6A	109.5
O1—C1—C2	112.96 (18)	C3—C6—H6B	109.5
O1—C1—H1A	109.0	H6A—C6—H6B	109.5
C2—C1—H1A	109.0	C3—C6—H6C	109.5
O1—C1—H1B	109.0	H6A—C6—H6C	109.5
C2—C1—H1B	109.0	H6B—C6—H6C	109.5
H1A—C1—H1B	107.8	O4—Cl1—O7	110.68 (15)
N1—C2—C1	113.07 (18)	O4—Cl1—O5	111.2 (2)
N1—C2—H2A	109.0	O7—Cl1—O5	112.8 (2)
C1—C2—H2A	109.0	O4—Cl1—O6	109.3 (2)
N1—C2—H2B	109.0	O7—Cl1—O6	108.2 (2)
C1—C2—H2B	109.0	O5—Cl1—O6	104.49 (19)
H2A—C2—H2B	107.8		

### Hydrogen-bond geometry (Å, °)

**Table:**

*D*—H···*A*	*D*—H	H···*A*	*D*···*A*	*D*—H···*A*
O1—H1···O5	0.89	2.02	2.860 (4)	157
N2—H2···O1^i^	0.83	1.98	2.803 (3)	169
C1—H1B···O2	0.97	2.52	3.126 (3)	121
C6—H6B···O7^i^	0.96	2.52	3.441 (4)	161

Symmetry codes: (i) *x*, −*y*+3/2, *z*+1/2.
